# FDM-Based 3D Printing of Polymer and Associated Composite: A Review on Mechanical Properties, Defects and Treatments

**DOI:** 10.3390/polym12071529

**Published:** 2020-07-10

**Authors:** Sachini Wickramasinghe, Truong Do, Phuong Tran

**Affiliations:** 1 Department of Civil & Infrastructure Engineering, RMIT University, Melbourne, VIC 3000, Australia; s3758687@student.rmit.edu.au; 2 College of Engineering and Computer Science, VinUniversity, Hanoi 14000, Vietnam; v.truongdt@vingroup.net; 3 CIRTECH Institute, Ho Chi Minh City University of Technology (HUTECH), Ho Chi Minh City 70000, Vietnam

**Keywords:** FDM, thermoplastic polymer, fibre-reinforced composites, defects, heat treatments, ultrasound-assisted printing, 3D printing, additive manufacturing

## Abstract

Fused deposition modelling (FDM) is one of the fastest-growing additive manufacturing methods used in printing fibre-reinforced composites (FRC). The performances of the resulting printed parts are limited compared to those by other manufacturing methods due to their inherent defects. Hence, the effort to develop treatment methods to overcome these drawbacks has accelerated during the past few years. The main focus of this study is to review the impact of those defects on the mechanical performance of FRC and therefore to discuss the available treatment methods to eliminate or minimize them in order to enhance the functional properties of the printed parts. As FRC is a combination of polymer matrix material and continuous or short reinforcing fibres, this review will thoroughly discuss both thermoplastic polymers and FRCs printed via FDM technology, including the effect of printing parameters such as layer thickness, infill pattern, raster angle and fibre orientation. The most common defects on printed parts, in particular, the void formation, surface roughness and poor bonding between fibre and matrix, are explored. An inclusive discussion on the effectiveness of chemical, laser, heat and ultrasound treatments to minimize these drawbacks is provided by this review.

## 1. Introduction

**Figure 1:**
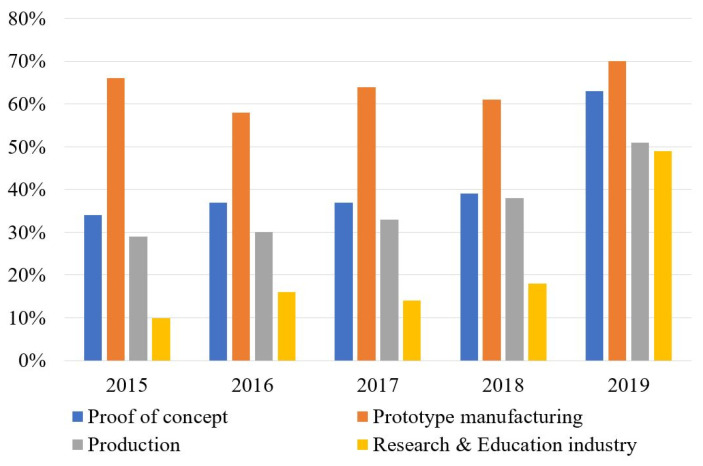
The percentage distribution of additive manufacturing (AM) from 2015 to 2019 in prototype manufacturing, production, the research and education industry and mechanical part manufacturing [1].

3D printing is a continuously evolving additive manufacturing (AM) technology that enables printing of lightweight and complex structures, which are hardly achievable by other manufacturing methods. The distribution of AM throughout 2015 to 2019 in prototype manufacturing, production, the research sector and mechanical part manufacturing is graphically shown in Figure 1. It is clearly visible that 3D printing has been mainly used in prototype manufacturing throughout all these years, and the growth of this technology in actual production is still at a lower level.

With the invention of different types of AM technologies, this method has spread to the medical, aerospace, automotive, food and engineering industries due to many advantages such as the production feasibility of complex designs, cost effectiveness, short lead times and repeatability [2,3,4]. The medical industry has started to experiment with developing tissues, organs and cellular structures using 3D printing [5,6]. By adopting 3D printing in the automotive industry, the time required for tooling is reduced, as well as the cost of manufacturing prototypes [7,8]. It has made it possible to develop unique parts for high end-low volume manufacturing [9]. The aerospace industry has become one of the most benefitted industries by 3D printing as conventional structures can be replaced by lightweight, flexible and improved geometrical structures to reduce fuel consumption and material waste [10,11].

**Figure 2:**
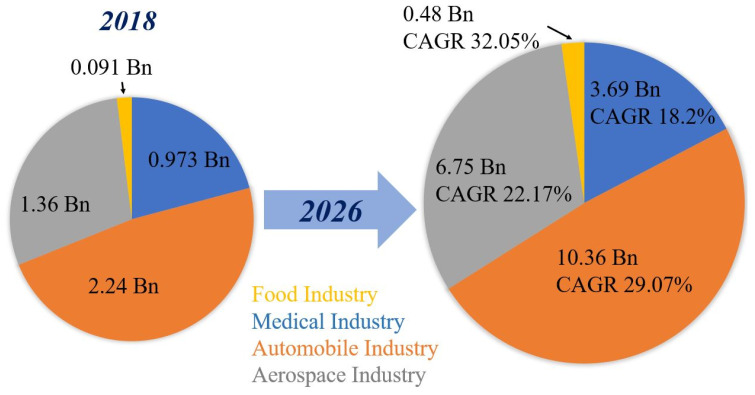
Compound annual growth rate (CAGR) of 3D printing in the food, medical, aerospace and automobile industries from 2018 to 2026 [12,13,14,15].

According to statistical data of 2018, it is predicted that the compound annual growth rate (CAGR) of 3D printing in the medical, automobile, aerospace and food industries will be 18.2%, 29.07%, 22.17% and 32.05% by the beginning of 2026, and this is graphically represented in Figure 2.

The materials used in AM include polymers, metals, ceramics and composites that could be in the forms of a semi-liquid, a liquid or a powder depending on the type of 3D printing process [2,16,17,18,19,20]. Since the 1980s, a wide range of 3D printing methods has been invented due to the high demand by manufacturing industries, and those can be categorized as stereolithography (SLA), selective laser sintering (SLS), laminated object manufacturing (LOM), solvent cast direct writing (SC-DW) and fused deposition modelling (FDM). The popularity of 3D printing is continuously enhancing due to its commercial and sustainable advantages such as the reduction of material input and repairability, the ease of the make-to-order process, energy savings and extended product life cycle [21,22,23,24,25,26].

Stereolithography (SLA) was the first 3D printing method to be developed, and the structure is built by using ultraviolet (UV) light-curable resins. The design is generated by a guided UV laser beam, which causes the resin to harden according to the given design and build each layer [27]. The surface finish quality of SLA printed parts is higher compared to other 3D printing methods. 

The selective laser sintering (SLS) method is a powder-bed-based printing technology. In this method, a laser beam moves along a predetermined path and sinters the powder to build the solid print [28,29]. When the laser beam hits the powder particles, they get fused due to the heat generated by the laser, and each printed layer is attached to the previous layer by the same fusing process [30]. 

In the laminated object manufacturing (LOM) method, sheets are cut using laser or mechanical cutters according to the required shape, then they are laminated together, or the sheets can be laminated together first and then cut into the required shape. The machining and material cost is lower compared to the other processes and can obtain a better surface finish in the final part [4]. In the solvent cast direct writing (SC-DW) method, a dissolvable polymer is combined with a rapid solvent evaporation process to print complex structures at room temperature [31]. 

Fused deposition modelling (FDM) uses a heated nozzle to convert the thermoplastic filament into a semi-molten form, which is extruded to build the structure through layer-by-layer deposition [32]. This method is further explained in Section 2, and it is the main 3D printing method we are focusing on in this paper. 

**Figure 3:**
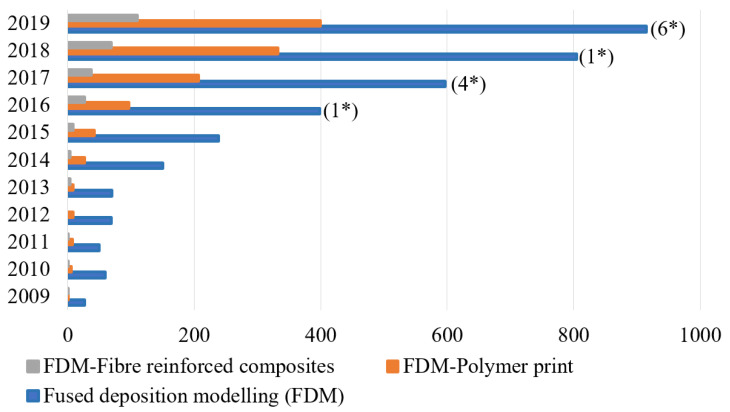
Number of publications on: FDM, FDM-polymer prints and FDM-fibre-reinforced composites from 2009 to 2019. The number within the brackets indicate the number of review papers on FDM-fibre-reinforced composites. Data obtained from the Web of Science.

Compared to other AM methods, as the FDM method offers many advantages including cost effectiveness, now, many researchers are moving towards FDM to study this process thoroughly. Figure 3 indicates the number of research publications relevant to the FDM process from 2009 to 2019. It demonstrates the growing interest towards the fibre-reinforced composites printed with this FDM technology. Even though a considerable amount of research work has been done in FDM polymer printing, the number of research works on FRC is much lower compared to that.

As FDM becomes a revolutionary manufacturing technology, the requirements from various industries start to escalate, and the demands of those industries are fulfilled. Printer manufacturing companies such as Stratasys, Ultimaker, Markforged, XYZprinting, Zortax, German RepRap and Dagoma are constantly engaging in improving the printers, while software companies such as Autodesk, Siemens PLM software and Dassault systems are developing new CAD software to be compatible with the new printers. Even after decades of development, there are still various problems related to printing, quality assurance and consistency in FDM, which are caused by undetected defects or imperfections in printed samples.

**Figure 4:**
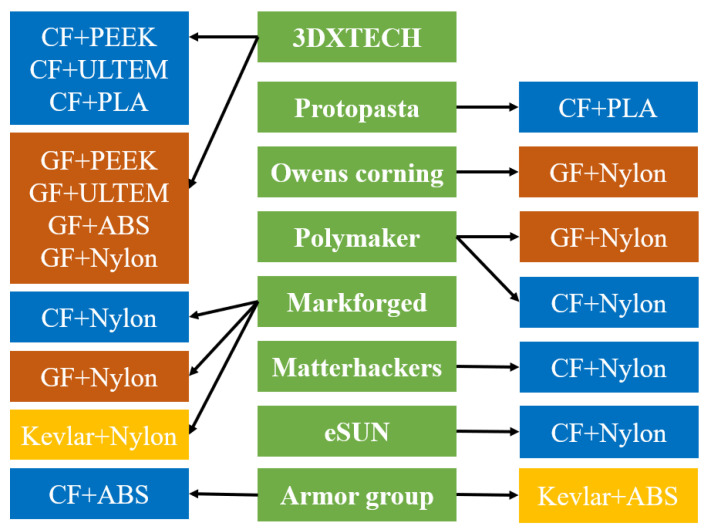
FDM filament and printer manufacturers (middle column) and their associated fibre-reinforced composite products. Key fibres reinforcements include carbon fibre (CF), glass fibre (GF) and Kevlar (KF), while popular matrix are polyether ether ketone (PEEK), Acrylonitrile butadiene styrene (ABS), Polylactic Acid (PLA) and Nylon.

Hence, this paper will focus on reviewing, characterizing and classifying defects and exploring different technical methods to reduce or eliminate them, in order to achieve better properties in 3D printed polymers and composites via FDM. The second section of this paper discusses the FDM process and thermoplastic polymer printing. The thermoplastic polymers that are discussed in this review are used as the matrix material in developing fibre-reinforced filament. Figure 4 exhibits the combination of matrix and fibre materials developed by various filament manufacturers. The middle column shows the filament manufacturer, while the other two columns show the fibre and matrix combination.

The third section is focused on fibre-reinforced composite printing using FDM. The fourth section explains the defects presented in FDM printed polymers and FRC that affect the improvement of the mechanical properties in printed parts. Finally, in the fifth section, different types of treatments to overcome these defects are thoroughly elaborated. 

## 2. Fused Deposition Modelling of Thermoplastic Polymers

**Figure 5:**
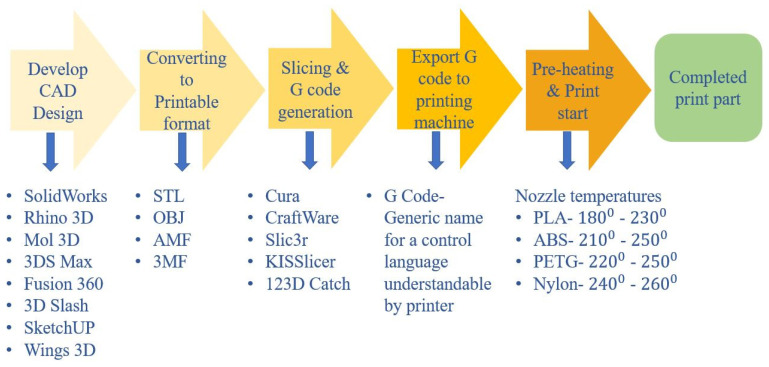
Simplified process flowchart of FDM including different types of software used in the 3D printing industry. (STL- Stereolithography file, OBJ- Wavefront 3D Object File, AMF-Additive manufacturing file, 3MF- 3D manufacturing format).

FDM is one of the widely used and rapidly evolving 3D printing methods compared to other additive manufacturing processes [33]. The simplified process flowchart of the FDM process is shown in Figure 5. FDM is a slow printing process, which is limited to materials with a low melting point. Furthermore, the print dimensions are restricted, and it gives a rough surface finish to the printed part [34]. 

It also brings about many advantages over these drawbacks, such as high reliability, a wide range of low-cost filament material availability and low maintenance and initial investment cost [35,36]. 

The FDM method can be utilized to print polymers, polymer matrix composites (PMC), bio-composites or polymer ceramic composites (PCC), nanocomposites and fibre-reinforced composites (FRC) [37]. ABS (acrylonitrile butadiene styrene), PLA (polylactic acid), Nylon/polyamide, ASA (acrylonitrile styrene acrylate), PET (polyethylene terephthalate), PETG (polyethylene terephthalate glycol-modified) and PC (polycarbonate) are the most commonly used polymers, while PEEK (polyether ether ketone), PEKK (polyetherketoneketone) and ULTEM (polyetherimide) are known as high performance polymers, and TPE (thermoplastic elastomers) is the newest flexible polymer material used in FDM. There is a wide range of reinforcement materials that can be incorporated into the FDM process, such as CNT (carbon nanotubes), graphene, copper, iron fillers, continuous and short fibres of carbon, glass and Kevlar. Further information on FDM polymer printing and fibre-reinforced composite printing emphasising carbon, glass and Kevlar is discussed in the next section. As polymer serves as matrix binding reinforcements in a composite material, our discussion begins with an explanation of thermoplastic polymer printing via FDM. The thermoplastic polymer matrix plays a major role in FDM fibre-reinforced composites. Both matrix and fibres have a great influence on the mechanical properties and the defects of the composite. Hence, it is important to have comprehensive knowledge of thermoplastic polymer prints before exploring fibre-reinforced composites. 

In fibre-reinforced composites, ABS, PLA and nylon are the widely used matrix material. The main reason for ABS, PLA and Nylon to be the most common types of thermoplastic filaments used in FDM is because of their low melting temperature. Due to the low strength and functional properties in pure polymer printed parts, these are mainly used to build concept samples or prototype samples. Much research has been conducted to identify these polymers’ behaviour during printing, microstructural alignment of the filament and the mechanical performance of FDM printed polymer parts under tensile, flexural and impact loading. 

### 2.1. Acrylonitrile Butadiene Styrene Polymer

ABS is widely used in toy manufacturing and household item manufacturing as it is known to have relatively low harmful effects on humans compared to other polymer materials [38,39]. Due to the presence of styrene in the chemical structure of ABS, it is not recommended to use it in medical implants as it lasts longer in the human body [40,41]. Since ABS exhibits better heat, chemical and moisture resistance, it is widely used in developing testing samples [42]. 

According to an experiment conducted by Dawoud et al. [43], it was observed that since there is no pressure applied during the process of FDM, the final parts contain more void regions inside the printed structure. These void regions can be minimized by employing a smaller layer thickness as it enhances the bond between layers, which reduces the interlayer distortion that causes micro voids in the structure [44,45,46,47,48,49,50]. When printing ABS using the FDM process, the process parameters such as infill density, orientation, layer thickness, airgaps, raster angle and width play important roles in providing better strength to the print part [51,52].

Despite the above-mentioned parameters, the strength of the ABS parts is affected by the nozzle diameter of the FDM machine. With an increased nozzle diameter, the strength between the layers is enhanced due to the reduction of voids, which positively impacts the tensile strength of the printed parts [43,53,54]. 

### 2.2. Polylactic Acid Polymer

When ABS is compared with PLA, it can be observed that even though ABS demonstrate better impact strength, the tensile strength is higher in PLA. Under proper conditions, PLA can easily degrade while other polymers are disposed or recycled [55,56]. The plasticity and toughness of parts made from PLA do not decline for a long period of time. 

Similar to ABS FDM, many research works have been conducted to identify the optimal conditions to achieve better mechanical properties for PLA as well. It is noted that tensile strength is mainly affected by the raster angle followed by the raster width and layer height. Most of the research works have confirmed that increasing the layer height of the print generates many voids in the microstructure, which reduces the tensile strength of the print part [57,58,59,60,61,62]. When the raster angle is at 0°, the tensile load is mainly borne by the PLA filament oriented in the longitudinal direction, and at the 90° raster angle, the load is carried by the bond between layers. Hence, for 90° rasters, the failure occurs due to the delamination and breakage of the layers, while in the 0° orientation, the failure occurs due to filament breakage [63,64,65,66]. 

**Figure 6:**
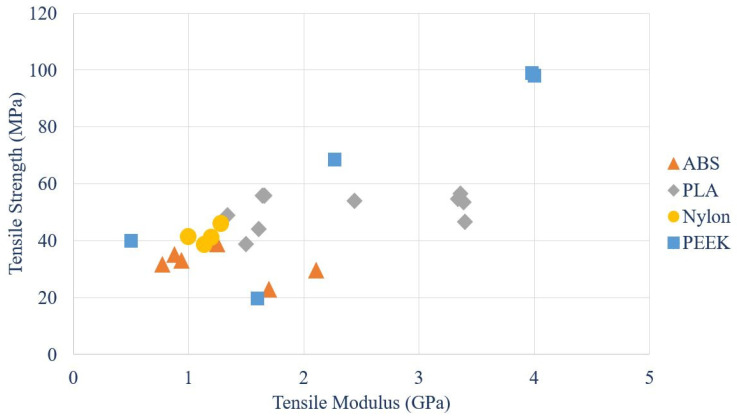
Tensile strength vs. modulus outspread of ABS, PLA, Nylon and PEEK specimens printed via FDM. Data obtained from [44,46,49,50,51,56,60,64,66,67,68,69,70,71,72,73,74,75,76,77].

By gathering data from different experiments, a comparison of tensile strength vs modulus of ABS, PLA, Nylon and PEEK polymers are given in Figure 6.

When considering all these results acquired by many research works, it is evident that the printing parameters greatly affect the mechanical properties of PLA printed parts. 

### 2.3. Polyamide/Nylon Polymer 

When compared with ABS and PLA, Nylon exhibits better chemical resistance and a higher tensile strength and Young’s modulus [78]. Some of the main advantages of FDM printed Nylon parts are high tensile and impact strength, good resilience and low creep. This material also achieves better mechanical properties at elevated temperatures, as the bonds between layers become much stronger at higher temperature [79]. Nylon is known to be a hydrophilic material, which negatively impacts the mechanical properties of printed parts due to moisture absorbency. The studies that have been conducted to identify the mechanical properties and the effect of different parameters associated with Nylon are limited compared to ABS and PLA [80,81]. Similar to ABS and PLA, it is noted that with the decrease of layer thickness in the printed part, the tensile strength is enhanced as the bond between layers is much stronger [82]. The most popular Nylon material that is used in the 3D printing industry is Polyamide 12 (PA 12). The crystallinity of Nylon is the main reason for it to achieve better functional properties such as mould shrinkage and chemical, wear and thermal resistance. Due to these benefits, this material is widely used in manufacturing home appliances and white goods, as well as aerospace and automotive engineering applications [83,84,85]. 

### 2.4. Polyetheretherketone Polymer

Other than the above explained polymers, there is another set of polymers used in the 3D printing industry known as high performance polymers or engineering polymers. PEEK is one of the widely used high performance polymers, which belongs to the PAEK (polyaryletherketone) polymer category. PEEK is a colourless, semicrystalline, organic thermoplastic polymer used in the engineering industry to build aircraft, rockets, racing cars and drone parts, which require excellent properties [86]. Up to now, only a few experiments have been conducted to identify the effect of print orientation, nozzle diameter, printing speed, extrusion speed, nozzle temperature and the infill density on the mechanical properties of PEEK [74,75,87,88,89,90]. Due to the inherent qualities of PEEK, it can be used to print parts for medical applications with better reliability [91,92]. The most popular areas that use PEEK in the medical industry are bone tissue engineering, orthopaedic implants, joint replacements, spinal implants, prosthesis systems and dentistry [93,94,95,96]. 

According to several research data, the distribution of the tensile strength vs. modulus of ABS, PLA, Nylon and PEEK is represented by a scatter plot, shown in Figure 6. As per the scatter plot, it is apparent that the tensile strength of PEEK is noticeably higher than PLA, ABS and Nylon. 

Even with the development of high performance polymers, the mechanical properties of polymer prints are low when compared with other manufacturing methods. As a solution, researchers have started experiments by adding various materials with better functionalities to the pure polymer, expecting to enhance the mechanical properties. With that, the development of composites by utilizing the FDM process has begun, and a detailed discussion on composite manufacturing is given in the following section.

## 3. Fibre-Reinforced Composite Printing 

A composite is a combination of different materials that are used to develop a material with improved functional properties. The main reason to develop composite materials in the 3D printing industry is to achieve better mechanical properties and various optical, thermal and electrical functionalities that are not attainable by pure polymer [97,98,99,100]. In a composite, one or more materials act as the reinforcing element, while another material acts as the matrix or binding material. There are various reinforcement materials that are available for FDM printed composites to obtain the required functional property, or a few different properties from the same composite. Depending on the necessity, particles, fibres or nanomaterial can be added to the polymer to print a polymer matrix composite (PMC). The most popular PMCs are micro- or nano-particle reinforced composites, metal particle reinforced composites and short or continuous fibre-reinforced composites [101,102,103,104]. By adding metal particles such as iron, copper, stainless-steel, titanium or nanoparticles such as carbon nanotubes, graphene or graphite to the polymer, a high-performance composite with embedded thermal, electrical, optical and excellent mechanical properties could be developed [105,106,107,108,109,110,111,112,113,114,115,116]. 

FDM printed composites have been employed in many industrial applications including the aerospace, automobile, marine, sports equipment, electrical and medical industries [117]. NASA and Piper aircraft are two major figures in the aerospace industry that use FDM to print manufacturing tools, functional prototypes, concept models and some complex lightweight parts [118]. In the automobile industry, FDM is mainly employed in printing jigs, fixtures and prototypes for testing. Team Penske is one of the most popular American pro car racing teams that uses FDM carbon fibre/Nylon 12 composites to print prototypes and end use parts for their IndyCar and NASCAR race cars [119]. Most of the medical industry applications are still under investigation for the biocompatibility of FDM printed composite parts, and experiments have been done to incorporate these composites to manufacture orthopaedic and dental implants [120]. The most widely used reinforcement material in PMC is short or continuous fibre reinforcements. This is mainly due to the high strength-to-weight ratio that can be achieved by fibre reinforcement and, also, because of the rigidity and corrosion resistance that are inherited in these composites [121,122,123,124]. This section, hence, focuses on elaborating research works that have been conducted relating to the fibre-reinforced composites printed using the FDM process. The fibre reinforcement could be either a synthetic, high performance fibre or a man-made or natural cellulose fibre. The selection of fibre reinforcement is totally dependent on the expected properties from the composite; hence, choosing the correct fibre type is a major issue in fibre-reinforced composite (FRC) printing. Carbon, glass and Kevlar are known as the extensively used high performance fibres, while flax, basalt, jute and bamboo are some of the rising natural fibres in the FDM composites industry [125,126,127,128]. The following sections discuss in depth both short and continuous fibre-reinforced composites, emphasising their properties, advantages and applications. 

### 3.1. Short Fibre-Reinforced Composites

**Figure 7:**
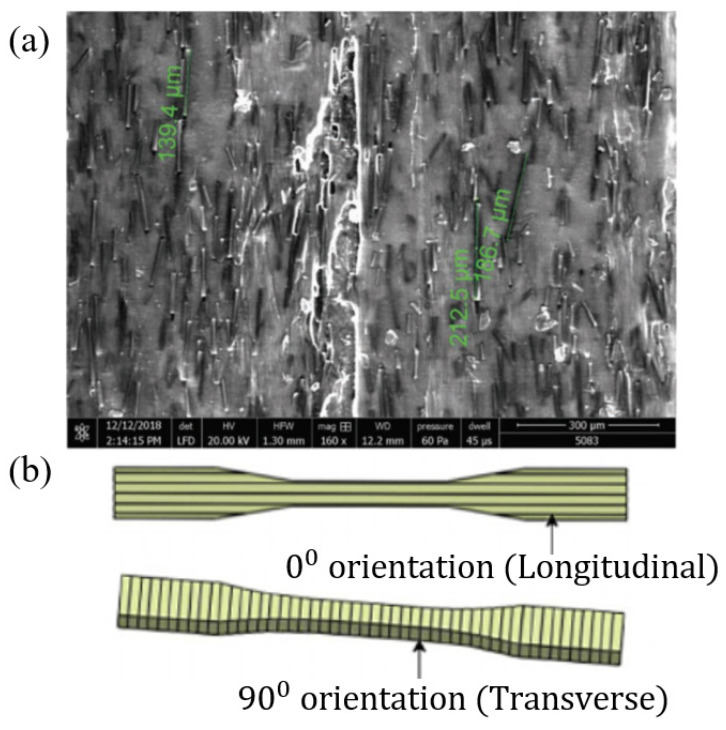
(**a**) Short CF immersed in Nylon matrix (onyx filament) [148]. (**b**) The orientation of the printed tensile specimens [152].

The main purpose of adding short fibres as the reinforcement of the polymer is to enhance the strength of the printed part due to the weak strength exhibited by the pure polymer printed parts, which restricts its applicability in industrial applications. Generally, the fibre-reinforced filaments used in the FDM process are manufacturing by mixing fibres into a molten thermoplastic polymer [129]. The arrangement of these short fibres within the filament is random, and this phenomenon is represented by Figure 7a, where short CF are immersed in Nylon matrix (onyx filament). When producing a fibre-reinforced filament, it is critical to control the fibre orientation, the mixture percentage of fibre and the optimal size of the fibre to prevent unnecessary complications such as the extruder clogging during printing. These parameters greatly affect the mechanical properties of the final print [130]. When compared to the pure polymer prints, the strength, stiffness, fatigue and corrosion resistance and damage tolerance of FRCs are considerably higher [125]. These mechanical properties can be further enhanced by employing the most suitable raster angle, layer thickness, infill pattern, number of reinforced layers, extrusion temperature of the filament, airgap, diameter of the filament, the time between adjacent layer deposition and the conditioning temperature [131,132,133,134,135,136]. 

Many research works have been conducted using carbon, glass and Kevlar fibres as these are identified as high-performance fibres that could potentially enhance the mechanical properties of FDM printed parts. The attraction towards the natural fibres such as flax, jute, basalt, bamboo and hemp are also increasing due to their eco friendliness and sustainability [137,138,139,140,141,142]. Most of the experiments have been performed with carbon fibre (CF) due to its excellent mechanical properties, lower density, corrosion, wear and moisture resistance, good thermal conductivity and electrical properties, low thermal expansion and piezoresistive behaviour, which is a special feature inherited from CF [143,144,145,146,147,148]. Hence, composites printed using CF as the reinforcement with matrix materials such as PEEK, PC, PE and PA are widely used in developing industrial applications, especially in the aerospace and automotive industries [149]. 

As per the findings of Ning et al. [150], it has been established that adding short fibres to a polymer material could improve the tensile strength and Young’s modulus, but it negatively impacts the ductility, toughness and yield strength of the specimen. To study these phenomena, CF/ABS composite specimens have been printed to perform mechanical tests. The perfect amount of fibre weight percentage and length of the short fibre were pointed out as 5 wt% and 150mm as the tensile strength and flexural strength of the specimen with 5 wt% CF were enhanced by 22.5% and 11.82%. When the fibre content was increased, this led to severe porosity in the microstructure of the printed part, causing poor mechanical strength. Hence, it is extremely important to identify the optimum levels of fibre percentage and the size of the fibre to improve the mechanical properties of the printed parts. To study the effect of adding short fibres to the pure polymer, Li et al. [151] performed an investigation by incorporating short CF in to the PEEK polymer matrix. Two specimens were printed, one in the horizontal direction and the other in the vertical direction. It was found that the vertically printed specimen had better flexural strength and modulus compared to the horizontally printed specimen. It was, however, evident that the strength of both specimens was increased with the addition of CF to the polymer.

To identify the effect of printing orientation, layer thickness and the printing temperature, Ding et al. [152] carried out an experiment using a CF/PLA composite printed using the FDM process. The orientation of the specimens is present in Figure 7b for better clarification. From the results, it was evident that specimens printed with a 0° fibre orientation obtained higher tensile strength than the specimens printed with a 90° orientation This was due to the way these two orientations bore the tensile load. For the 0° fibre orientation, the tensile strength of the specimen was mainly dependent on the strength of the CF, while in the 90° orientation print, the strength depended on the adhesion between layers. The tensile and impact strength of the 0° orientation prints decreased with the increase of the layer thickness, whereas in the 90° orientation print, the strength increased. Furthermore, it was observed that with the increment of printing temperature, the strength of the 90° orientation print specimen was enhanced as the bond between layers became stronger and due to the reduction of the porosity. With further temperature increment, the strength reduced as the PLA was degraded. Ultimately, it was confirmed that fibre orientation, layer thickness and printing temperature parameters had major impacts on the FDM printed composites.

Spoerk et al. [153] observed that adding CF could enhance the flexural strength and modulus of pure PP (polypropylene) printed samples. Furthermore, it has been noted that a 10% volume fraction of CF in the PP matrix illustrated better surface quality, good extrudability and less agglomeration of material, better printability, strong adhesion with the matrix and good dispersion of fibres compared to a 15% and 20% volume fraction. Similar findings were observed by Liao et al. [154] when they conducted a study using a CF/PA 12 composite printed with the FDM process. The flexural, tensile and impact properties of the PA12 print specimen were notably enhanced by adding 10 wt% of CF to it. Hence, with the above results, it was clear that adding short fibres to the pure polymer could greatly impact the mechanical properties of the printed parts. After identifying the effect of incorporating short fibres into the pure polymer, researchers have tended to perform experiments to clarify the optimal parameter conditions, which are mentioned above, to obtain a composite material with enhanced mechanical properties. 

A similar experiment was conducted by Magri et al. [155] to investigate the effect of infill and the printing temperature of CF/PLA composite. With that, the optimum nozzle temperature and the print orientation to achieve higher tensile strength was obtained as 230 °C and [0°, 15°, −15°]. Despite all the above-mentioned mechanical property improvements, it is clear that adding short fibres increases the porosity of the printed part, preventing it from achieving the maximum strength. According to Zhang et al.’s [156] research, it was identified that adding short CF to the ABS matrix increased the porosity of the printed part compared to the pure ABS polymer print. This scenario was also confirmed by the experiment conducted by Tekinalp et al. [157].

**Figure 8:**
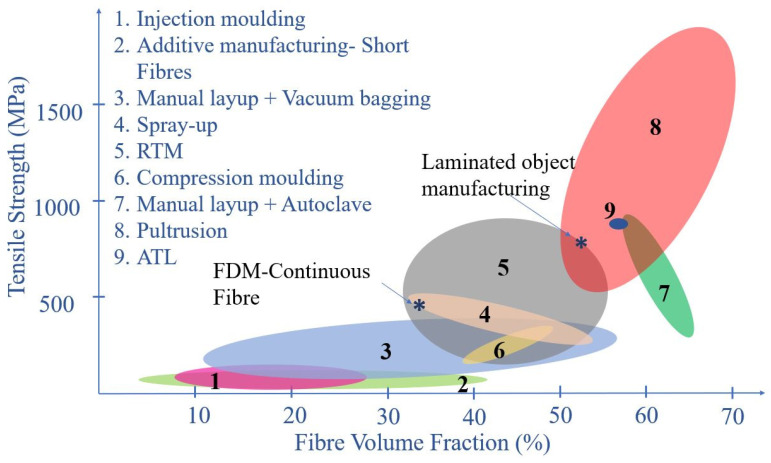
Tensile strength vs. fibre volume fraction comparison of short fibre-reinforced composites with respect to conventional composite manufacturing methods [98] (RTM: resin transfer moulding, ATL: automated tape laying).

Hence, it is understandable that adding short fibres as the reinforcement to the thermoplastic polymers could affect the print part in both a positive and negative manner. From all the test results, it is evident that the mechanical properties of these composites are enhanced compared to the pure polymer print, but still, they are way behind with respect to the mechanical properties of the conventionally manufactured composite materials. This scenario is graphically depicted in Figure 8. Even with the increment of the fibre volume fraction, the tensile strength of short fibre-reinforced composites lies below 500 MPa, while many other conventional composite manufacturing methods yield higher values of tensile strength.

Due to this reason, researchers have tried to incorporate continuous fibres into the polymer matrix to attain higher strength from FDM printed composites. In the following section, the attempts made by various researchers to improve the mechanical properties of continuous fibre-reinforced composites printed via the FDM process are thoroughly discussed. 

### 3.2. Continuous Fibre-Reinforced Composites 

**Figure 9:**
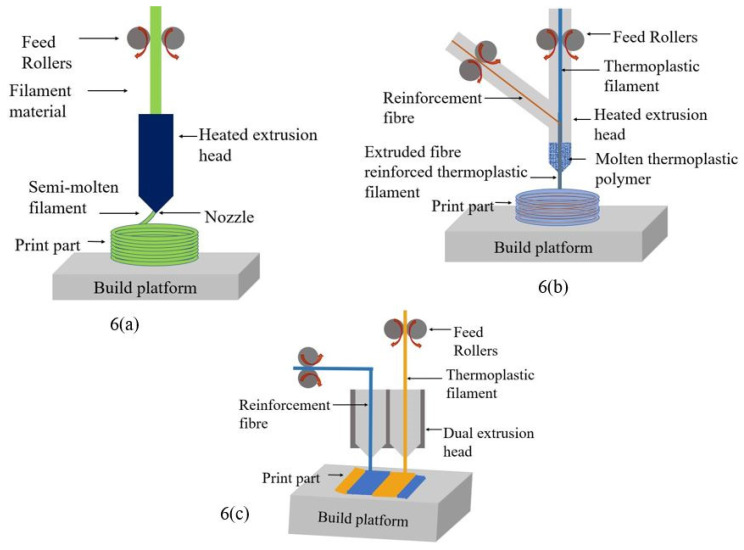
(**a**) Common FDM printer for polymers and short fibre-reinforced composites (SFRC). (**b**) Co-extrusion FDM printer for continuous fibre-reinforced composites (CFRC). (**c**) Dual-Extrusion FDM printer for CFRC.

In continuous fibre-reinforced composites (CFRC), the composite can be printed by a dual extrusion [158,159,160] or co-extrusion [127,161] method. In co-extrusion, the thermoplastic resin filament and the fibre filament are separately supplied to the FDM print machine head. The thermoplastic filament gets molten inside the heated nozzle, and when the reinforcing fibre is passed through the nozzle, it gets impregnated by the resin. Once the resin covered fibre is extruded through the nozzle and deposited on the printing platform, the extruded filament gets attached to the previous layer and solidifies [162,163]. In the dual extrusion method, the reinforcing fibre filament and the thermoplastic resin filament are separately extruded through two nozzles onto the printing plate. A schematic diagram of co-extrusion and dual extrusion is presented in Figure 9b,c, respectively, while a common FDM print process is presented in Figure 9a.

As the fibre is always aligned with the printing direction in CFRC, it is possible to control the fibre orientation. Due to the tension exerted by the reinforcing fibre, nozzle clogging can be eliminated during printing [164]. Carbon, glass and Kevlar (aramid) are majorly used in CFRC due to their better functionalities, which are beneficial in high performance applications [165]. Before the development of commercial printers to print continuous fibre-reinforced composites via the FDM process, various experiments had been conducted by researchers to identify a suitable method to incorporate continuous fibres into thermoplastic matrix. The majority of the experiments are based on building new extrusion heads and attaching them to an existing FDM printer [126,166,167]. Matsuzaki et al. [161] modified the extrusion head of a commercial FDM 3D printer with a preheating apparatus. In this method, the reinforcing fibre tow is directly fed into the nozzle without any additional feeding mechanism while the PLA filament is supplied through a gear and stepping motor mechanism. As the diameter of the nozzle is smaller than the diameter of the thermoplastic filament, once the filament is melted into a resin, the solid filament pushes the resin through the nozzle, and the reinforced filament deposits onto the platform in a layer-by-layer arrangement. The tensile strength of continuous carbon fibre-reinforced thermoplastic (CFRTP) and continuous jute fibre-reinforced thermoplastic (JFRTP) composites are noted as 185.2 MPa and 57.1 MPa, which are higher than the PLA values. SEM images exhibit fibre pull-out in the fracture surface of both CFRTP and JFRTP and void spaces, which are the most common drawbacks in every fibre-reinforced composite.

Similarly, Bettini et al. [165] performed a modification to the extrusion head to develop a continuous aramid fibre-reinforced composite. A commercial 3D printer was modified by adding a feed roller for continuous fibres and a die with a diameter of 1mm. The pulling force generated during the deposition caused the reinforced fibre to extrude through the nozzle. In this method, a better deposition and rate of solidification were achieved by controlling the deposition speed and the layer thickness. Duigou et al. [168] modified a commercial 3D printer by adding a filament cooling system and a nozzle with a flat head and used flax fibres as the continuous fibre reinforcement in a thermoplastic composite. The tensile test results indicated an improvement in stiffness and strength of the fibre-reinforced specimen compared to pure PLA. By analysing the microstructure of the fracture surface, it was noted that failure occurred due to fibre breakage and fibre pull-out, which are the same causes for any synthetic fibre-reinforced composite. 

**Figure 10:**
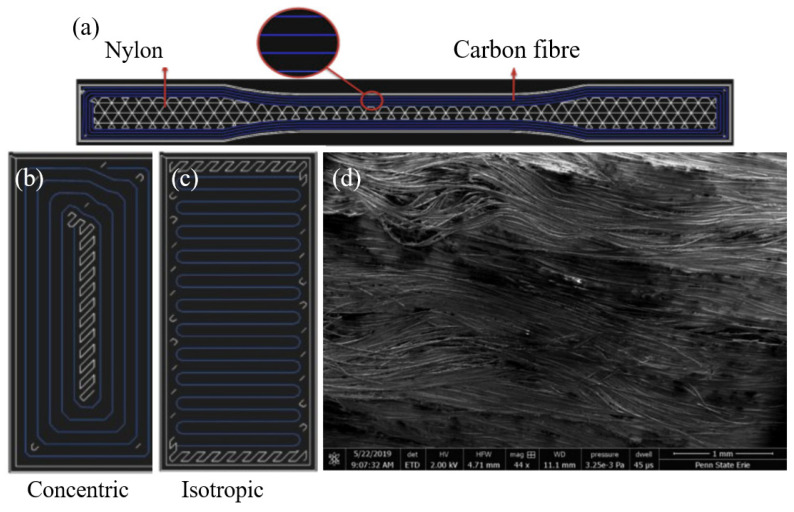
(**a**) Continuous carbon fibre and thermoplastic nylon filament in the tensile specimen [176]. (**b**) Concentric infill. (**c**) Isotropic infill [175]. (**d**) Continuous carbon fibre strands in a composite [148].

In 2014, the first commercial printer that was able to print continuous carbon fibres was released as Mark One, and later, in 2016, the Mark Two was introduced to the market. Both machines contain the same working principle, where one nozzle extrudes the thermoplastic filament while the other nozzle supplies the reinforcing fibre [169,170]. With that, research has expanded to identify optimal conditions to print continuous fibre-reinforced composites to obtain higher mechanical properties that are unattainable by polymer or short fibre-reinforced composites [158,171]. Figure 10a indicates the graphical representation of the reinforcing fibre and the thermoplastic filament in a tensile specimen, while Figure 10d shows the continuous fibre strands in a fibre-reinforced composite. By investigating the mechanical properties of continuous carbon fibre-reinforced composite printed with the Mark One printer, Blok et al. [172] observed that even if the tensile and flexural strengths increased by incorporating a continuous fibre, the compressive strength was lowered. This was due to high void content and/or due to poor bonding between the interfaces of fibres and matrix. By further investigation, it was noted that fibreless areas were filled with the nylon matrix, and the unfilled areas were left as voids. Both nylon fill areas and voids caused weakening in the printed composites, as well as the non-uniform distribution of the fibres. 

A similar experiment was conducted by Frank van der Klift et al. [173] with Nylon matrix and CF, and it was identified that, though the tensile strength was enhanced with fibres, adding more fibre layers increased the void content, causing a poor tensile modulus. Caminero et al. [174] performed Charpy impact tests on carbon, Kevlar and glass continuous fibre-reinforced composites. It was identified that an increased fibre volume fraction resulted in higher impact strength, and an on-edge orientation performed better compared to a flat orientation. The printing orientation, fibre volume fraction (FVF), impact strength and microstructural analysis are given on Table 1. The highest impact strength was displayed by the Kevlar fibre-reinforced flat oriented specimen with the maximum fibre volume fraction. It can be noted that with the increment of FVF, both the on-edge and flat orientation samples improved their strengths, but the on-edge samples exhibited excellent strength compared to the flat orientation samples. From the SEM images, it was evident that bonds between layers and the fibres in each layer bore the impact load in the flat orientation, while separate layers of fibres endured the load in the on-edge orientation. 

Araya-Calvo et al. [175] performed an experiment to analyse the flexural and compressive properties of continuous carbon fibre-reinforced thermoplastic composites and the effect of reinforcement distribution, print pattern and orientation on these mechanical properties. The details related to the experiment are given in Table 2, and the images of the concentric and isotropic infill pattern are given in Figure 10b. The highest compressive and flexural modulus were obtained from the design with the concentric infill pattern, and also, it was confirmed that both compression and flexural modulus improved by maximizing the fibre volume fraction. By the SEM image observation, it was verified that having nylon layers in between fibre-reinforced layers increased the bond within layers while reducing the delamination during compression. With the incorporation of a variety of continuous fibres including natural and synthetic fibres, studies to analyse the impact of infill density, fibre orientation and type of fibre on mechanical performances of FDM printed composites have been conducted by both experimental and modelling methods [175,176,177,178,179,180]. Mohammadizadeh et al. [181] performed an investigation to study the effect of temperature, fibre type, infill density and fibre orientation on continuous carbon, glass and Kevlar fibre reinforcement composites printed on the Mark Two printer. The highest tensile strength was exhibited by the CF specimen with the combination of both isotropic infill and two concentric rings. The scanning electron microscope (SEM) images clearly explained the main reasons for the tensile test failure as fibre pull-out.

With the experiment of Akhoundi et al. [182], they noticed that a continuous glass fibre-reinforced composite with PLA matrix exhibited higher tensile strength, with a 49% fibre volume fraction and rectangular infill pattern. To study the effect of Nylon fill and the tensile properties of 3D printed composites with different concentric rings and layers, Hui Mei et al. [183] conducted research on continuous fibre-reinforced composites of carbon, Kevlar and glass. They developed five types of specimens with different combinations. It was indicated that the tensile strength and elastic modulus increased with the increment of concentric rings and the number of layers. A CF reinforced specimen with eight layers and eight rings demonstrated the highest tensile strength and modulus, while the lowest strength and modulus were exhibited by the Kevlar fibre reinforced specimen and Glass fibre reinforced specimen, respectively. By analysing the effect of Nylon fill, they confirmed that the highest tensile strength and modulus of the composite were exhibited by the rectangular fill, whilst the lowest tensile properties were shown by the hexagonal and triangle infill. All the details relevant to this experiment are given in Table 3 for further clarification. 

Lash et al. [184] carried out an experiment to identify the mechanical properties of continuous carbon fibre-reinforced composites printed on the Markforged X7 printer with a much stiffer matrix named onyx. This stiffness was achieved by including a microfibre reinforcement of carbon into the Nylon matrix. The maximum tensile strength was obtained by 0° oriented specimens followed by 90° oriented specimens and 45° oriented specimens. Similarly, Dong et al. [185] also achieved the highest tensile strength in the 0° orientated continuous Kevlar fibre-reinforced composite, which was considerably higher than the short Kevlar fibre-reinforced composite. 

These continuous fibre-reinforced thermoplastic composites (CFRTC) have expanded into many engineering applications. This is due to their particular features such as low weight, long life span, high strength and low maintenance, which are difficult to achieve in current engineering materials without performance trade-off. Due to the better orientation and interface area of continuous fibres, the continuous fibre-reinforced composites are much stronger and stiffer than short fibre-reinforced composites. According to many research works, it has been identified that continuous fibres can improve the tensile strength and Young’s modulus of the composite at the expense of ductility and toughness, but the improvements in strength and Young’s modulus are significantly higher. The improvement in the mechanical performance of continuous fibre-reinforced composites is considerably higher than the short fibre-reinforced composites. With proper printing parameters and the elimination of defects such as the porosity and poor bond between fibre and matrix, the mechanical properties of these composites can be improved to compete with the mechanical properties of conventional composites [186].

As per the above discussion, it is evident that FDM printed fibre-reinforced composites are already being incorporated in many industries due to their prevailing advantages. The research sector is continuing experiments to enhance the properties of these composites, as they have the potential to replace conventional materials. In the future with the development of commercial FDM printers that have the ability to position the fibre and matrix in different combinations, it will facilitate the printing of FRC with further improved mechanical properties [187]. By introducing electrical and magnetic properties to fibres and matrices, FDM could print complex FRC structures having multiple functionalities [188]. For FDM to be a long-established manufacturing process of FRC, it is essential to develop sustainable and recyclable fibres and matrices that are compatible with the FDM process. These are some of the future aspects of fibre-reinforced composite printed FDM, but to strengthen the future of these composites, it is crucial to overcome the existing drawbacks. 

Common defects presented in FDM print polymers and composites are discussed in the next section including suitable treatments to minimize those drawbacks. 

## 4. Common Defects in FDM Printed Polymers and Fibre-Reinforced Composites

**Figure 11:**
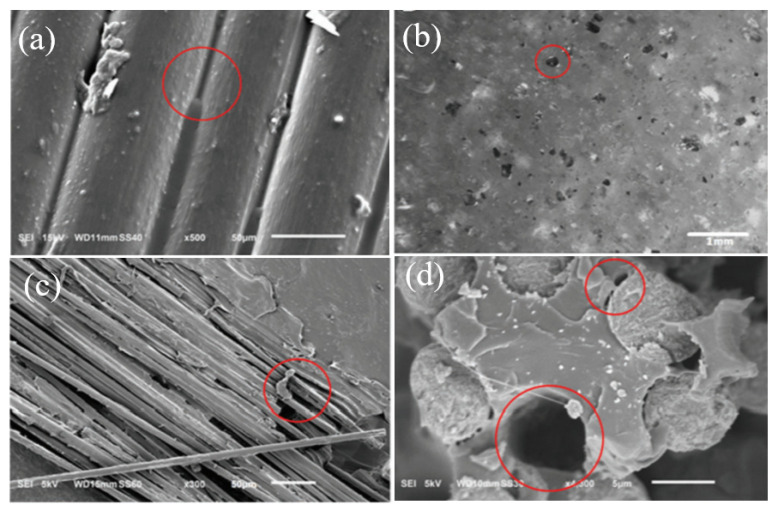
(**a**) Surface roughness of a Nylon sample. (**b**) Micropores on the surface of a PLA sample. (**c**) Poor resin infusion into fibres. (**d**) Fibre pull-out from the matrix [189].

As per many research works, it has been clearly identified that there are a few drawbacks in FDM printed polymers and fibre-reinforced composites that cannot be rectified only by engaging optimal printing parameters. These drawbacks directly affect the strength and appearance of the printed part. Generally, these defects can be summarized as shape distortion that occurs due to residual stresses caused by non-uniform temperature gradients, micro voids in the matrix and filaments, uneven fibre distribution within the fibre-reinforced thermoplastic filament, poor bonding between fibres and matrix and surface roughness occurring due to the staircase effect. Oztan et al. [189] identified these defects in fibre-reinforced FDM print composite through SEM image analysis. The most prominent defects were the surface roughness and porosity on the top surface of the print and the poor bond between fibre and matrix. The SEM images of those defects are given in Figure 11.

Figure 11a shows the surface roughness where the nylon filaments are separated in a line-by-line arrangement, which indicates the unsmooth surface, and in between those lines, gaps are visible. Figure 11b exhibits micropores on the surface of the PLA printed parts. Figure 11c indicates the poor infusion of nylon resin into the carbon fibre bundle, and Figure 11d indicates the poor bond between carbon fibres and nylon matrix as fibres have pulled out from the matrix. By the experiment of Papon et al. [190], again it was noted that voids, poor bonding between fibre and matrix and gaps between beads and within layers affected the fracture toughness of those fibre-reinforced composites. The porosity or voids are created in different ways; they can be generated during printing, or the voids can be already present within the filament, especially in fibre-reinforced filaments. During printing, voids can be developed due to air traps in the matrix, gaps between the beads and layers, uneven matrix distribution and uneven filament diameters. 

**Figure 12:**
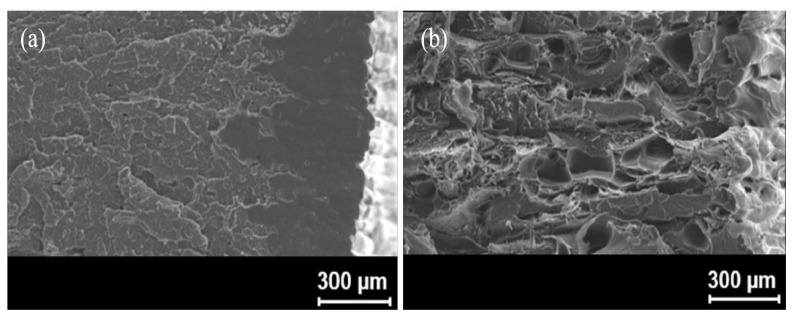
(**a**) ABS print cross-section with a layer thickness of 0.06 mm. (**b**) ABS cross-section with a layer thickness of 0.17 mm. The increased layer thickness caused many voids [53].

Some of the void formation in FDM is inevitable due to the nature of this printing process. The voids between the layers are much bigger and differ with the airgap and layer thickness of the print, while the voids present inside the filament and matrix are much smaller and difficult to control by changing printing parameters [191]. According to many experiments, it has been identified that the gaps between the layers, which contribute to the failure of the printed part by delamination, can be reduced by minimizing the layer thickness [192,193,194]. Figure 12a,b shows the effect of layer thickness on the void formation, and it is evident that the voids between layers could be minimized by reducing the layer thickness. The layer thickness in FDM typically lies within the range of 0.05 mm–0.4 mm. This process can print finer layers compared to SLS as the finest layer thickness in SLS is 0.08 mm, but it cannot reach up to the finest layer height of SLA, which is 0.025mm [195]. Even though a finer layer thickness minimises the void content in the FDM printed part, it negatively affects the production time. When the layer thickness is small, the number of layers required to complete the part increases; hence, the production time will also increase. Several experiments have observed many void formations within the extruded filament, which were smaller than 16.4 μm, and they greatly affected the porosity percentage in the printed part [196,197]. All above-mentioned voids act as a failure initiation point when a load is applied to these samples. When the stress is concentrated on those weak void areas, it causes premature failure in the specimen, and this void content mainly affects the transverse properties of the specimen, which are dominated by the matrix strength [189,198,199].

He et al. [197] identified a large distribution of voids near the crack initiation point and recorded poor resistance to crack growth within those void areas. These micro voids are one of the main reasons for the poor strength exhibited by parts printed with the FDM process. Aside from the mechanical properties, the porosity in the FDM print parts also negatively affects their sealing functionality. With several experimental results, it can be observed that these parts exhibit poor sealing of liquids and gases, which restrict their usage in sealing applications [200,201].

As discussed in previous sections, the strength of the printed part is increased with the incorporation of fibres into the pure polymer. It is evident that adding fibres increases the void content in the microstructure of the print part. When the thermoplastic filament is reinforced with the fibres, the uneven distribution of fibres results in the creation of porous areas within the fibre.

**Figure 13:**
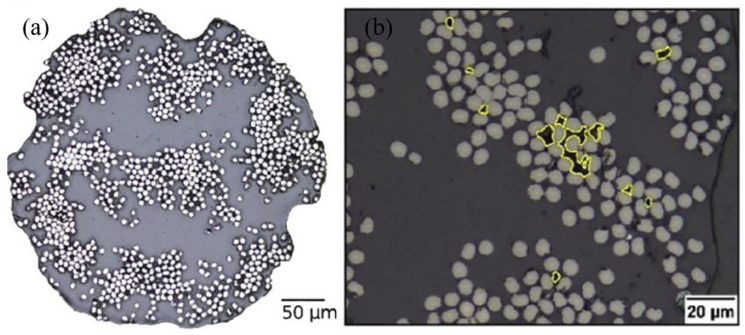
(**a**) Cross-section of continuous CF reinforced filament. (**b**) Magnified image representing voids between fibres [202].

**Figure 14:**
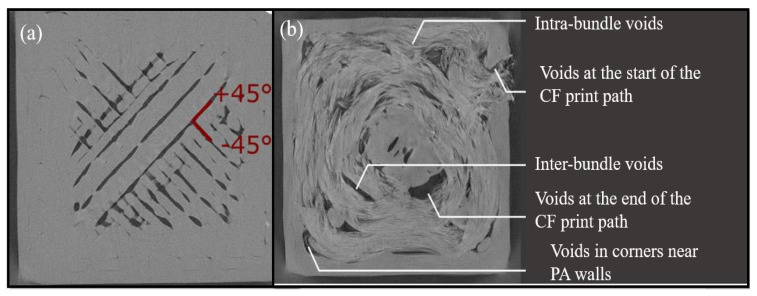
(**a**) Porosity in between beads of PLA. (**b**) Increased porosity within CF bundles, inside the CF bundle, beginning and at the end of the CF print and near the walls of PLA [203].

Kabir et al. [202] analysed the cross-section of CF reinforced filament and GF reinforced filament and noted that there were fibre-rich and matrix-rich areas. Due to the poor impregnation of the matrix in fibre-rich areas, the porosity within the filament increased. Figure 13a indicates the cross-section of CF reinforced filament, and Figure 13b indicates the void areas within fibres in yellow colour. Soete et al. [203] confirmed that the amount of large void areas with irregular shapes started to increase with the increment of the carbon fibre layers. When the part was printed with pure polymer, the voids were visible only in between the beads, which are indicated in Figure 14a. Once the fibres were introduced, porosity in between the fibre bundles, within the fibre bundles and at the beginning and end of the print were clearly visible, e.g., shown in Figure 14b. It has also been noted that these void regions cause the rapid initiation and propagation of cracks in between the fibre layers. When FDM printed FRC are subjected to tensile loading, the failure mainly occurs due to fibre breakage. By analysing the fracture surfaces of the FRC tensile specimens, it is visible that fibre pull-out is also one of the reasons for the failure [204,205].

In FRC, when the bond between fibres and matrix are not strong enough, fibre pull-out occurs easily. To enhance the bond, the fibres should be properly coated with the matrix. If the wettability of the fibre is poor, the bond with the matrix becomes weaker due to poor impregnation [206]. This is another noticeable drawback in the FDM process. 

**Figure 15:**
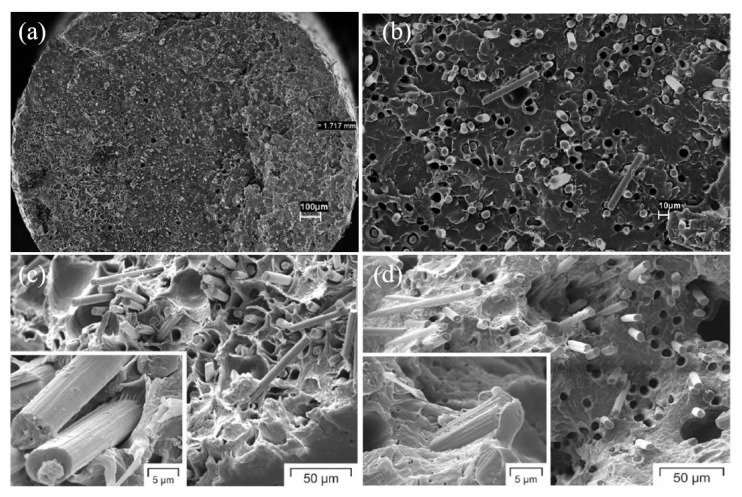
(**a**) Cross-section of CF reinforced PLA filament. (**b**) Magnified cross-section of CF reinforced PLA filament. (**c**) Cryo-fractured surface of 15% CF reinforced PP composite. (**d**) Cryo-fractured surface of 20% CF reinforced PP composite. (**a**,**b**): [69], (**c**,**d**): [153].

**Figure 16:**
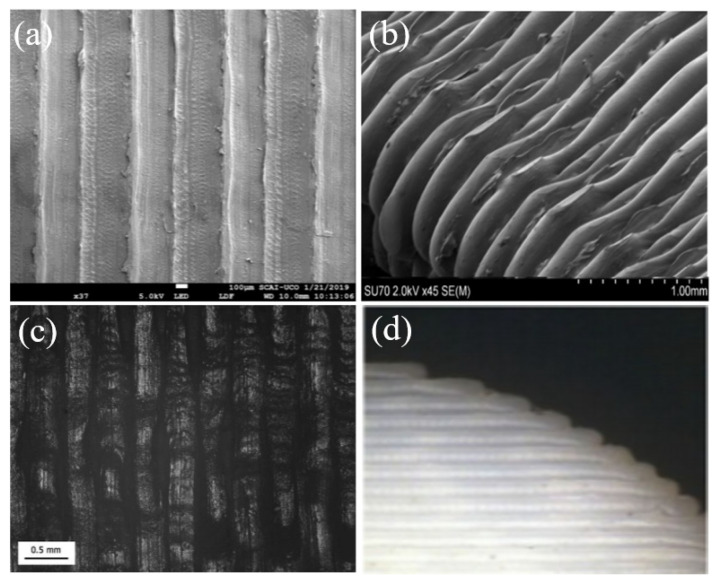
(**a**) Surface roughness of the Nylon FDM specimen [207]. (**b**) Surface roughness of the ABS FDM specimen [208]. (**c**) Surface roughness of the PLA FDM specimen [209]. (**d**) Staircase effect in the FDM printed curved surfaces [210].

The fractured surface indicates visible voids created due to fibre pull-out. It also indicates that fibres have easily pulled out as there was no considerable damage done to the matrix. The cross-section of CF reinforced PLA matrix is shown in Figure 15a,b, while the cryo-fractured surface of 15% CF and 20% CF reinforced PP is shown in Figure 15c,d. The other defect in FDM processed prints is the surface roughness on the part. The side-by-side line effect and the staircase effect that happen due to layer-by-layer deposition cause the surface roughness. This scenario is prominent in inclined and curved surfaces. Figure 16a–c indicates the surface roughness of Nylon, ABS and PLA printed using the FDM process.

Because of the line effect that has occurred during the filament deposition, the finished surface of these parts tends to be rough. As per Figure 16d, it is clearly visible that due to the layer arrangement process, the staircase effect is prominent in curved structures [207,209,211]. This is noted as one of the drawbacks in FDM printed parts when compared with the surface finish of parts created by milling or moulding [189].

Another prevailing defect in the FDM process is the internal stress built up in the part during printing, due to rapid heating and cooling cycles. This causes non-uniform temperature gradients, and the resulting residual stress leads to shape distortion. Rapid cooling enables the deposited layer to solidify quickly. When a newly extruded filament gets deposited on the solidified layer, it generates a local re-melting effect, ensuring the bond between solidified layer and the filament. This results in uneven heating and cooling, which produce non-uniform temperature gradients. Due to this non-uniform temperature gradient, uneven stress builds up in both the previously deposited layer and the newly deposited layer. These stresses affect the shape and dimensions of the final parts. There are different types of distortions, such as transverse or longitudinal shrinkage, bucking, twisting, or angular distortion. This shrinkage causes delamination of layers and warping, where the part becomes curved from the corners and unsticks from the printing platform [212,213,214]. The shape distortion can be minimized by employing the optimal nozzle temperature, a slower printing speed, a 45° raster angle and increased layer thickness [215,216]. The most common method to prevent warping is applying an adhesive to the printing platform so that the part sits tightly on the platform without unsticking. Placing rough borosil glass on the printing bed, applying Kapton tape, which is made from polyamide film and silicone adhesive, treating the printing platform with polyvinyl acetate (PVA)-based compounds, enclosing the printer with an insulation casing, reducing the infill and designing the bottom layer of the part so that it can compensate for the stress are some of the common practices to minimize the warping [217]. 

Currently, various methods have been developed to minimize these drawbacks. They can be either pre-processes or post-processes, where the treatment could be applied before printing or after the printed part is completed. A discussion elaborating on those treatment processes is given in the next section. 

## 5. Treatment Methods to Overcome or Minimize the Defects in FDM Print Polymers and FRC

Different methods have been developed by researchers to overcome the drawbacks in FDM print parts, which are mentioned in the above section. The use of chemical solutions, heat, laser and ultrasound to enhance the properties of those printed parts are discussed in this section.

### 5.1. Chemical Treatment Process

**Figure 17:**
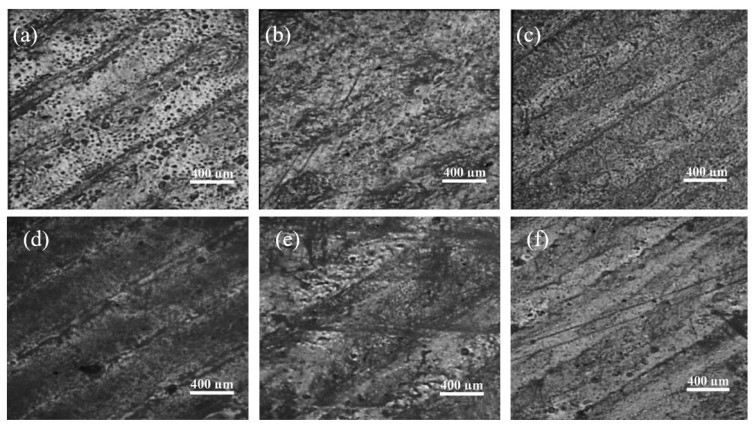
SEM images of ABS specimens treated with acetone: (**a**) 3 min, (**b**) 5 min and (**c**) 7 min. ABS specimen treated with dichloroethane: (**d**) 3 min, (**e**) 5 min and (**f**) 7 min. The raster lines disappear, making the surface smooth [219].

Chemical treatments are incorporated to enhance the surface quality of the print parts and to modify the surface of fibres to improve the bonding with the matrix. The most common chemical used to reduce the surface roughness is acetone. The part can be immersed in the acetone solution or it can be treated with hot or cold acetone vapour. Immersion is less time consuming, and it does not produce harmful gases as in cold vapor processing; also, it is much less expensive compared to hot vapor processes. Hambali et al. [218] noted that, once the ABS printed part was immersed in the acetone solution, the surface roughness was reduced by 97.2%, and the surface became shinier than in the original ABS part. The tensile strength of the specimen reduced by 42.58% due to the reaction between the solution and filament. A similar experiment was conducted by Jayanth et al. [219], where ABS specimens were immersed in acetone and dichloroethane solutions. The surface of the specimen treated with dichloroethane became much smoother than the one treated with acetone. The reduction of surface roughness was within 60%-91% of that for the unprocessed surface, while the height reduction was within 60%-93%. In both cases, the tensile strength decreased by around 48% compared to the original samples. Figure 17a–c shows the SEM images of ABS samples treated with acetone, and Figure 17d–f shows samples treated with dichloroethane for 3 min, 5 min and 7 min, respectively. It indicates that raster lines were gradually disappearing with the treatment time, making the surface smoother. According to Galantucci et al.’s [220] study, they also observed that an ABS part treated with 90% dimethyl ketone (acetone) and 10% water solution enhanced its flexural strength, as well as its surface smoothness. Other than immersing the print part in a chemical solution, many other investigations have used acetone vapor to study its effect on surface modification. As per those experiments, the surface roughness of parts printed with ABS, ABS+, PLA and PLA+ noticeably reduced their surface roughness values. Specifically, the staircase effect in ABS printed parts was almost removed as acetone dissolved ABS much more easily than PLA.

When acetone dissolves the outer surface of the print part, the voids on the surface and gaps between layers get filled with dissolved polymer, and the surface becomes smoother when solidifying. This process negatively affects the mechanical properties of the treated part [221,222,223,224,225].

**Figure 18:**
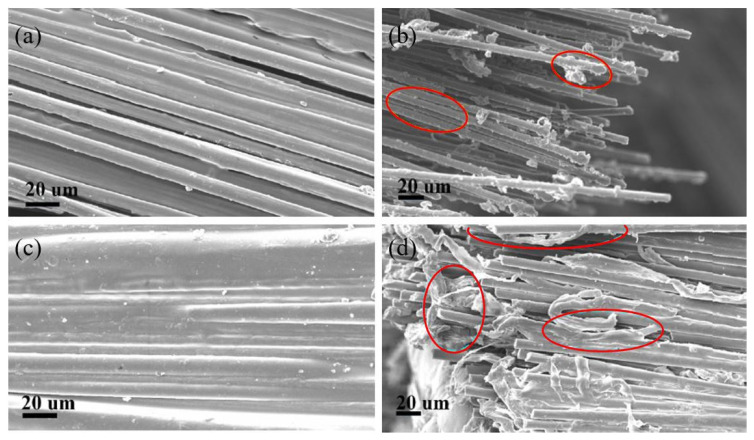
SEM images of CF reinforced PLA filament: (**a**) Untreated filament surface. (**b**) Fibre pull-out of the untreated specimen after the tensile test. (**c**) Chemical treated filament surface. (**d**) Fibre pull-out of the chemical treated specimen after tensile test with better deposition of the matrix on the fibre [126].

Apart from those chemical treatments on the surface, many other chemical treatments have been used to modify the surface of the fibres in fibre-reinforced composites. Researchers have followed various methods to enhance the wettability of the fibres so that the bonding strength between the fibre and matrix in FDM printed FRC can be improved while fibre pull-out is minimized. Li et al. [126] treated the carbon fibre (CF) reinforced PLA filament with methylene dichloride to dissolve the PLA, and then, the CF was modified with the use of emulsifying surface active and antifoaming agents. The final results showed better bonding between the CF and PLA matrix. From Figure 18a,c, it is clearly visible that treated CF was properly coated by the matrix and eliminated the gaps between fibres. Figure 18b shows that without treatments, fibres totally separated from the matrix during fibre pull-out. Figure 18d indicates that after modifying the fibre surface, the fibres were still coated by the matrix even after the pull-out during the tensile test.

Han et al. [226] showed that combining silane coupling agents with plasma to treat CF reinforced PP could improve the interfacial bond between the fibre and matrix. With the introduction of many functional groups on CF, the chemical bond between the fibre and matrix became stronger. Hence, the interlaminar shear strength of the treated sample was improved. Li et al. [227] observed that, when the short CF was treated with HNO_3_^,^ the strength of the bond with ABS and polyamide-6 matrices increased. CF was submitted to oxygen plasma treatment by Montes-Moran et al. [228], and they identified that the bond between CF and the PC matrix was considerably improved, causing higher interlaminar shear strength. This was because of the increment of functional groups on the CF surfaces by the plasma treatment and because the surface became smoother as the carbonaceous impurities on the surface cleared due to plasma oxidation. According to Li et al. [229], it was again noted that, when CF was treated with O_3_, the number of carboxyl functional groups on the surface increased, resulting in better adhesion between the fibre and polyamide-6 matrix. With that, the interfacial shear strength of the treated sample was enhanced by 60%. Another method that has been investigated by researchers is printing under different atmospheric conditions to overcome these drawbacks. Lederle et al. [230] processed the FDM print under nitrogen gas atmosphere and noted a significant improvement in elongation at break and a 30% increment in tensile strength for both ABS and PLA specimens. The better mechanical properties were achieved due to the reduction of polymer surface degradation and improved layer adhesion caused by oxidation process suppression.

Maidin et al. [231] printed an ABS sample by the FDM process in a vacuum chamber. Since there was no heat transfer occurring during the vacuuming process, the cooling speed of the printed layers was reduced. Hence, the surface became much smoother due to the absence of blobs and stringing. It was also noted that the staircase effect was significantly reduced while improving the surface smoothness by 9% compared to the normal print sample. With the above-mentioned treatments, it is noted that chemical treatment on printed samples can be mainly employed to enhance the surface smoothness. Although it enhances the flexural strength, it is not suitable when a higher tensile strength is required. The bond between fibres and matrix in a composite can be improved by chemically treating the fibre surface prior to mixing it with the matrix in developing the fibre-reinforced filament. The printing process being conducted under different atmospheric conditions could also help in improving the surface quality and mechanical properties of the printed sample. 

### 5.2. Laser Treatment Process

Another method that is used to improve the surface quality of the FDM print parts is by laser treatment. Many experiments have confirmed that exposing the surface of the printed part to a CO_2_ laser can improve its surface roughness. When the printed part is treated with a CO_2_ laser, the material is quickly heated and melts. The polymer gets sublimated by a photochemical ablation process and converts the solid directly to the gaseous state. Because of this process, the unnecessary irregularities on the surface are removed, and the surface becomes smoother. By a few experiments, it has been identified that, compared to PLA, the surface smoothness in ABS does not increase. Hence, this method is more suitable for PLA print parts [232,233,234]. As per Lambiase et al.’s [235] study, which was conducted with a 30 W power laser, when the laser paths overlapped, the surface became much smoother without generating any parallel grooves. Laser treatment eliminated the surface roughness by melting down the peaks and filling the pores on the surface with molten polymer [236]. Chen et al. [237] conducted laser treatment on a Cu fibre PLA composite and noted that not only the surface quality, but the mechanical properties also improved. The sample treated with a laser with a power of 5 W and having a laser beam diameter of 200 μm had a reduction of its surface roughness by 91%. The tensile strength and Young’s modulus increased by 25.6% and 34.1%, respectively, compared to the untreated sample.

**Figure 19:**
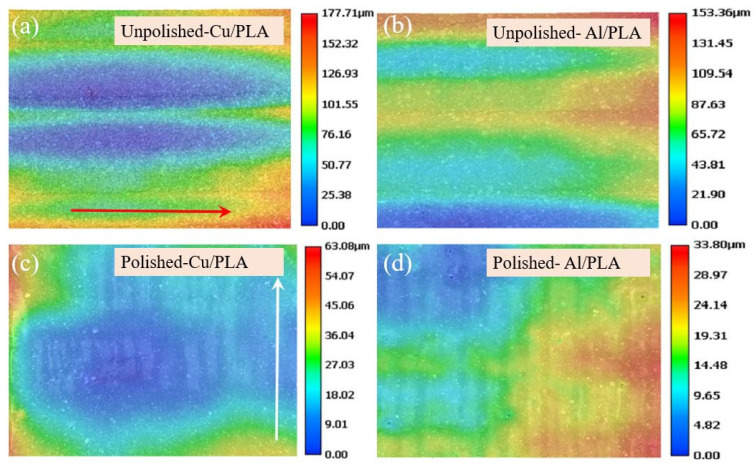
Surface topographies of: (**a**) unpolished Cu/PLA specimen; (**b**) laser polished Cu/PLA specimen with reduced surface roughness; (**c**) unpolished Al/PLA specimen; (**d**) laser polished Al/PLA specimen with reduced surface roughness. (**a**,**c**): [237], (**b**,**d**): [238].

This was due to the elimination of voids within the structure and improved bond strength between the fibre and matrix. The same results were also observed from the Al fibre-reinforced PLA composite printed with the FDM process. When the part was exposed to a laser beam, the polymer chains broke down, and the surface melted. The molten material filled the voids, and once the laser was removed, it solidified [238]. Figure 19a,c indicates the unpolished surface topographies of Cu/PLA and Al/PLA composites, respectively. Both of those images indicate irregular peaks, while Figure 19b,d indicates smoother surfaces of laser polished Cu/PLA and Al/PLA composites. 

With these results, it is evident that, by utilizing the optimal conditions of laser treatment, the surface quality and tensile properties can be significantly increased compared to chemical treatments. Recently, AREVO, a technology company based in California, developed a method to print fibre-reinforced thermoplastic composites with minimum void content. The method employs a laser heat source to melt the continuous fibre-reinforced thermoplastic filament, and then, it is compacted by a roller to build the 3D part. This technology would be highly beneficial in the aerospace, construction and transportation industries due to its capability of developing complex and high strength structures [239].

### 5.3. Heat Treatment Process: Annealing

**Figure 20:**
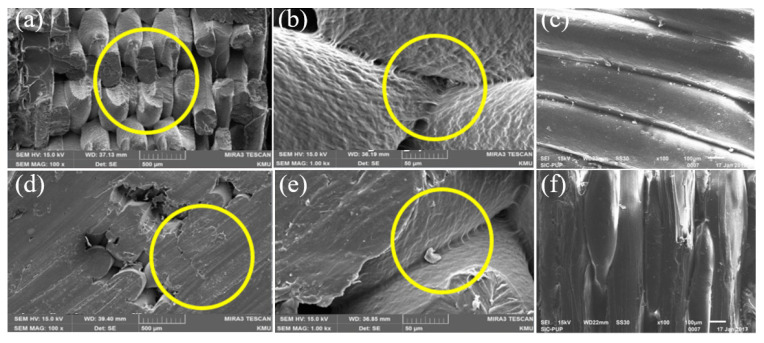
(**a**) Fracture surface of the untreated PLA specimen. (**b**) Bond between PLA filaments of the untreated specimen. (**d**) Fracture surface of the annealed PLA specimen. (**e**) Bond between PLA filaments of the annealed specimen. (**c**) Surface of the untreated ABS specimen. (**f**) Surface of the annealed ABS specimen. Heat treated specimens increased their layer and raster adhesion. (**a**,**b**,**d**,**e**): [243], (**c**,**f**): [242].

One of the most popular methods used to enhance the strength and surface quality of the FDM print parts is heat treatment or thermal annealing. This is a post-processing on complete prints where many investigations have been conducted to understand the effect of this process on the mechanical properties of polymers and composites. With several research works, it has been identified that thermal annealing increases the interlaminar toughness of polymers, making their performance better than injection moulding samples [240]. Singh et al. [241] found that when the ABS print part was treated with heat, the surface roughness value and staircase effect were significantly reduced. As the density of the part increased due to the heat, the gaps between layers filled, causing a smoother surface. Figure 20c,f exhibits the surface of untreated and heat-treated ABS specimen, and the SEM images show that the bonding between rasters was improved due to annealing. When the annealed temperature reached the glass transition temperature, ABS began to melt slightly. Due to the viscosity reduction at the glass transition temperature, the molecular surface tension was minimized, causing the ABS material to flow on the surface. The reflow of the material filled the porous areas, gaps and staircase effect within layers, resulting in a smoother surface finish and better mechanical properties. When the temperature increased from 105–125 °C, the tensile, flexural and impact strengths also increased, but the results of those mechanical properties were almost similar when the treatment time was increased from 20–30 min. It was, hence, confirmed that the annealing temperature had a huge impact on the final outcome, while the time duration of annealing was insignificant [242]. 

Several experiments have been conducted on PLA printed parts, as PLA is one of the most commonly used polymers in FDM. As per Hong et al.’s [243] work, it was noted that the mechanical properties including flexural strength and compressive strength were increased due to heat annealing. The bonds between the layers became much stronger with higher temperatures and longer exposure, whereas a sample treated at 140 °C for 600 s showed the maximum bond between layers. Figure 20a,b shows the fracture surface and bond between filaments in an untreated PLA specimen, while Figure 20d,e shows the fracture surface and bond between filaments in an annealed PLA specimen. From those images, it is clearly visible that heat treatment enhanced the bond between rasters and layers. Even though higher temperatures enhanced the strength of the part, the ductility was drastically reduced. Hence, it is advised to treat the sample with a low heat level to preserve the ductility while enhancing the strength [244]. Akhoundi et al.’s [245] study indicated several improvements in the printed structure of PLA after thermal annealing. XRD (X-ray diffraction) confirmed that once the PLA samples were annealed, the amorphous areas became semicrystalline. Microscopic analysis showed improved bonding between rasters and layers, as well as no visible voids in the microstructure. Wach et al. [246] noted that at higher temperatures, the crystallites of PLA grew smaller, while at lower temperatures, the crystallites were much bigger. In both scenarios, the flexural properties exhibited similar results. Hence, it was confirmed that the flexural strength could be increased up to 11%–17% even by keeping the part in a DSC (Differential scanning calorimetry) furnace at a 95 °C for 15 min. Aside from polymers, fibre-reinforced composites printed via FDM have also been identified to improve their mechanical properties when subjected to annealing.

While crystallinity occurs within the polymer, the bonds between the matrix and filler are also improved [247,248]. Bhandari et al. [249] observed that annealing could improve the interlayer tensile strength of short CF reinforced PLA and PETG printed composites. The tensile strength of both composites increased by two time and three times, respectively. Similar investigation was conducted by Rangisetty et al. [250] using short CF reinforced ABS, PLA and PETG. All the composite specimens were treated for 60 min under different temperatures due to the varying glass transition temperatures of the polymers. Annealing temperatures were noted as 65 °C, 110 °C and 85 °C for CF- PLA, CF-ABS and CF-PETG composites, respectively. The results indicated that the tensile strength increment of CF reinforced PLA, ABS and PETG was 16.8%, 3.34% and 12.4%, respectively. When considering the common results of these experiments, it could be identified that heat treatment increased the crystallinity in the polymer and improved the bonding between layer. This was the main reason for treated samples to have higher strength with slightly lower ductility [251,252]. Hence, it is important to treat the sample at the optimal temperature and time to improve the mechanical properties without compromising the ductility. 

### 5.4. Ultrasound Treatment Process

**Figure 21:**
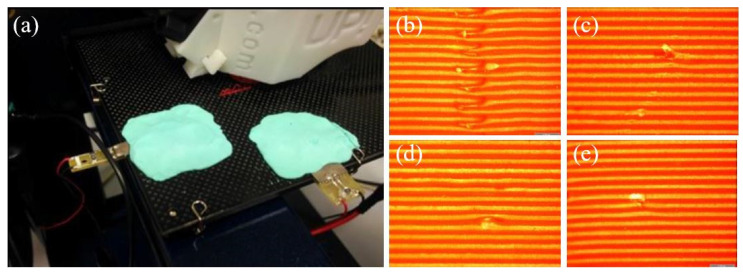
(**a**) Ultrasonic transducers mounted to the printing platform. (**b**) Surface of the original ABS sample. (**c**) The 11 kHz treated surface. (**d**) The 16 kHz treated surface. (**e**) The 21 kHz treated surface. The quality of the surface finish improves with the increment of the frequency [255].

Ultrasound is a growing treatment process for FDM printed parts, which can be applied before, during or after printing to improve the quality. This method has been utilized in various other industries to enhance the surface quality of products. As this is a non-chemical/non-thermal process, there are no adverse effects on the final product. Up to now, there have only been few experiments that have been conducted to investigate the effect of ultrasound on printed polymers and composites. In a few experiments, it has been identified that, by supplying ultrasonic vibration during printing, the surface quality of the final product can be improved while reducing the staircase effect and layer thickness [253,254]. Maidin et al. [255] performed an experiment to identify the impact of ultrasound vibration on the ABS polymer print by accompanying it with an ultrasound frequency of 11 kHz, 16 kHz and 21 kHz. The vibration was supplied by mounting piezoelectric transducers onto the printing platform where the vibration occurred horizontally during printing. Figure 21a presents the apparatus of the experiment, and Figure 21b–e shows the surface of the original specimen and the 11 kHz, 16 kHz and 21 kHz treated specimens.

**Figure 22:**
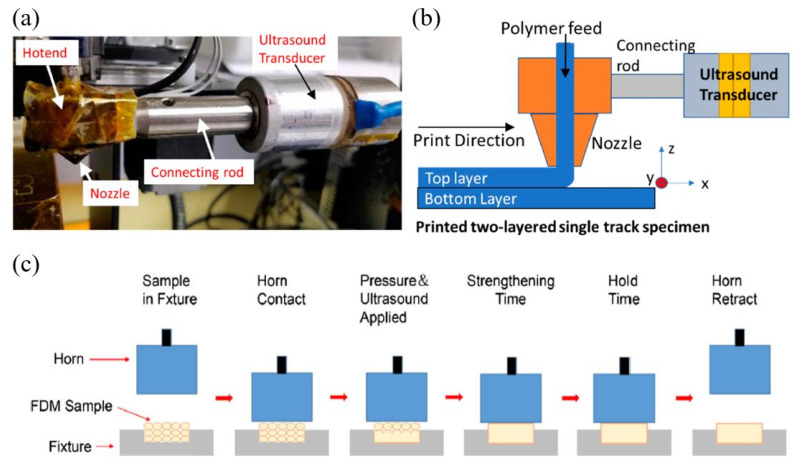
(**a**) Ultrasonic transducer attached to the printing head. (**b**) Schematic diagram of the apparatus. (**c**) Ultrasound horn setup. (**a**,**b**): [256], (**c**): [257].

The results confirmed that the sample treated with a 21 kHz frequency achieved an excellent surface finish. The layer thickness was also reduced by 0.02 mm, which implied that compression occurred while printing due to the vibration. Most importantly, the tensile strength of the treated specimens significantly increased. It was noted that the tensile strength could be further improved by providing higher ultrasound frequency. By the study of Tofangchi [256], it was identified that ultrasound vibrations had the capability of enhancing the layer adhesion of ABS prints. A piezoelectric bolt clamped transducer was connected to the heater block of the print head with the help of a stainless-steel rod and fasteners. The ultrasound frequency of 34.4 kHz was provided during the second layer deposition. Figure 22a,b illustrates an ultrasonic transducer setup and the schematic diagram of the apparatus for further clarification. The results of the trouser peel test indicated a 10% increment in the layer adhesion of the ultrasound treated specimen when compared to the untreated ABS specimen.

**Figure 23:**
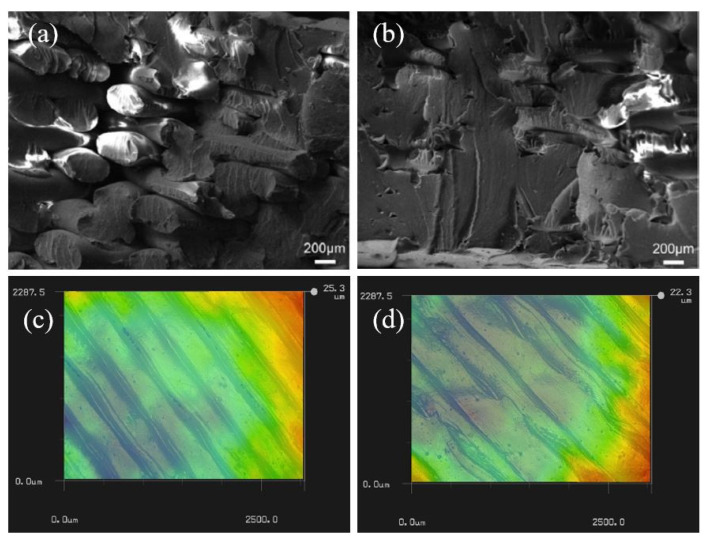
(**a**) Fracture surface of the untreated ABS specimen. (**b**) Fracture surface of the ultrasound treated ABS specimen with improved adhesion. (**c**) Surface morphology of the untreated ABS with visible stripes. (**d**) Surface morphology of the ultrasound treated ABS specimen. (**a**,**b**): [258], (**c**,**d**): [257].

As mentioned at the beginning of this section, ultrasound can be applied with pressure to the completed print part to enhance its mechanical properties. In several investigations, it has been observed that this is one of the successful methods to minimize the internal damages of the print parts and to enhance the mechanical properties. Wu et al. [258] conducted research to increase the interlayer shear strength and the bending strength of an ABS sample printed via the FDM process. Due to the inherent properties of FDM printed parts, the mechanical performance of those samples was restricted. In the results, it was observed that there was a 3% reduction of the thickness, improved dynamic mechanical properties and a 10.8% and 12.5% increment of bending strength and modulus due to the ultrasonic strengthening. The maximum bending strength of 69.26 MPa was obtained by providing a 20 kHz frequency with 3.5 kg/cm^2^ pressure for 0.65 s. SEM images indicated that the internal defects were repaired throughout the cross-section of the ABS part, and with the increment of the pressure up to 5 kg/cm^2^, the gaps between deposited layers were fully fused. The fracture surfaces of the original ABS specimen and an ultrasound treated specimens are shown in Figure 23a,b, respectively, and it is visible that the gaps between filaments fused together. A similar method was followed by Li et al. [257] with the exact same frequency, time and pressure condition to study the impact of ultrasound strengthening on the tensile properties and surface roughness of ABS printed parts using the FDM technology. The results indicated that both the tensile strength and Young’s modulus improved by 11.3% and 16.7% compared to the untreated specimen. A schematic diagram of the ultrasound setup used for this study is presented in Figure 22c.

**Figure 24:**
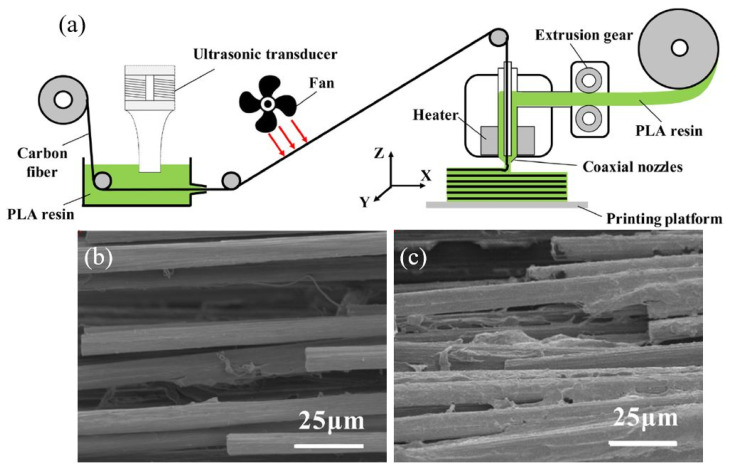
(**a**) Apparatus of the experiment. (**b**) Untreated CF bundle immersed in PLA resin. (**c**) Treated CF bundle immersed in PLA resin [260].

The internal fusing mechanism due to ultrasound vibration was explained as the transformation of ultrasound energy to friction energy, which then was converted into heat and deformation energies, causing fusion within the rasters. Figure 23c,d presents the surface morphology of untreated and treated ABS, and it is visible that ultrasound treatment smoothed the surface of the printed part as the stripes were gradually reduced. When ultrasound vibration is generated in a liquid, those vibrations generate cavitation and significantly improve the capillary effect [259]. This phenomenon can be beneficial in impregnating fibres in the resin and enhancing the bond between the matrix and fibres. Qiao et al. [260] investigated the effect of supplying ultrasound during the impregnation of PLA into the carbon fibre bundle. The apparatus of the experiment is given in Figure 24a for the ease of understanding. The carbon fibre bundle was passed through the PLA resin with a constant speed while keeping the ultrasonic transducer at a 10 mm distance from the bundle. The cavitation generated by ultrasound reduced the surface tension and the viscosity of the PLA, resulting in better coating around the fibres.

The SEM images indicated a proper distribution of resin into the fibre bundle, which led to a higher bond strength between CF and PLA. The SEM images of the untreated and ultrasound treated fibre bundles are given in Figure 24b,c, respectively. It was clear that ultrasound treatment improved the impregnation of PLA resin as the fibres were properly covered with the resin compared to the untreated bundle. The maximum tensile strength was obtained by the 15 mm/s treatment speed with a 40 μm ultrasonic amplitude, whereby both the tensile and flexural strength were enhanced by 34% and 29%, respectively. Relevant details for all the above-mentioned experiments in ultrasound treatment are given in Table 4 for the ease of understanding. The most important advantage of treating with ultrasound was that it did not cause any chemical reaction during the process. With these experimental results, it was evident that ultrasound could be a revolutionary treatment process for FDM print parts in enhancing their finished quality and mechanical properties. 

## 6. Conclusions

When considering the statistical data, it should be noted that the research sector related to AM (Additive Manufacturing) has grown from 10% to 50% since 2015 to 2019, which indicates that 3D printing is a rapidly growing manufacturing method. Medical, aerospace, automotive, food and engineering are the mainly benefitted industries with the development of 3D printing. Compared to available 3D printing methods, fused deposition modelling (FDM) has gained significant traction due to its high reliability, low initial investment and maintenance cost and the availability of low-cost materials. Up to now, a wide range of studies has been carried out to identify the effect of printing parameters such as raster angle, layer thickness and nozzle temperatures on the performance of printed polymers via FDM. The most common polymer materials that have been used are ABS, PLA and Nylon. Findings from several research papers can be summarized as below.

The optimal value of the raster angle to achieve the highest tensile strength in polymers varies, and it could be either 0° or 90°. Print strength depends on the strength of the thermoplastic filament and the bond strength between layers.

- Several experiments that have been conducted with short fibre-reinforced composites have resulted in determining a 10% volume fraction as the optimal level. They have also identified that the type of matrix in the composite also affects the mechanical properties.
- Researchers have developed two types of printing methods to print CFRC, namely co-extrusion and dual extrusion. In co-extrusion, fibre and thermoplastic filaments are separately fed into the nozzle where the fibre gets coated by the melted polymers inside the nozzle. In dual extrusion, the fibre and thermoplastic filament are deposited on the print platform via two separate nozzles.
- After analysing the fractured surfaces of both types of fibre-reinforced composites, it has been identified that failures mainly occur due to fibre pull-out, fibre-breakage and debonding, while the void formation inside the structures acts as weak areas, which initiate the failure.
- Fibre pull-out occurs due to poor adhesion between the matrix and fibre. Void formation could occur within the structure during printing or within the fibre-reinforced filament. Surface roughness occurs due to line-by-line deposition, and the staircase effect occurs due to layer-by-layer deposition. These are known as the most common drawbacks in FDM print parts.
- The above defects can be minimized or eliminated by chemical, laser, heat or ultrasound treatments.
- Chemical treatment can be used on the completed part to reduce the surface roughness, but this negatively affects the strength of the part. Some chemicals can be used to treat the fibre to increase the functional groups on the surface, which will react with the polymer to enhance the bond between the polymer matrix and fibre.
- When printing is conducted under a vacuum, the staircase effect can be eliminated, and when printing is done in a nitrogen atmosphere, both surface smoothness and the mechanical properties can be improved.
- Treating printed parts with a laser is also known to reduce the surface roughness values and improve the strength.
- Heat treatment or annealing is the widely used post-processing method to enhance the mechanical performance of the FDM prints. When heat is applied to the part, the layer-to-layer gaps are filled, causing a smoother surface. Due to the viscosity reduction at the glass transition temperature, the molecular surface tension is minimized, causing the material to flow on the surface. The reflow of the material fills the porous areas, gaps and staircase effect within layers, resulting in smoother surface finish and better mechanical properties.
- Ultrasound is the most recently used treatment method even though it has been around for a long time. Ultrasound can be employed in different ways to improve the quality of the print. This can be supplied to improve the impregnation of polymer into the fibre bundle and enhance the bond between them. Ultrasound transducers can be attached to the print platform to provide vibrations, which will reduce the staircase effect and improve the surface. Ultrasound can be provided to the finished part, where it will fuse the voids and gaps in the structure. The best advantage of ultrasound is that it does not cause any chemical reaction during the process. Hence, it is encouraged to conduct further studies related to ultrasound treatment that will benefit the mechanical performance.

As FDM is one of the best methods to build complex and lightweight structures, once its limitations are overcome, the manufacturing possibilities will be even more promising. 

## Figures and Tables

**Table 1:** Printing specifications, fibre volume fraction (FVF), impact strength and microscopic analysis details [174].

Printing Machine	Fibre + Matrix	Specimen Description	Test Specifications	Strength (MPa)	Microscope Analysis of Fracture Surface
Markforged- Mark Two	Carbon + Nylon	On-edge, 3.38% FVF, 0.125mm layer thickness	Charpy Impact-ASTM D6110	24.73 ± 1.61	On-edge: brittle fracture surface, void spaces, poor bond within layers and fibre breakage.
		On-edge, 24.82% FVF, 0.125mm layer thickness		59.76 ± 3.98	
		On-edge, 33.16% FVF, 0.125mm layer thickness		82.26 ± 6.79	
	Kevlar + Nylon	On-edge, 7.82% FVF, 0.1mm layer thickness		36.42 ± 1.28	
		On-edge, 29.53% FVF, 0.1mm layer thickness		95.11 ± 7.05	
		On-edge, 34.65% FVF, 0.1mm layer thickness		184.76 ± 15.11	
	Glass + Nylon	On-edge, 7.82% FVF, 0.1mm layer thickness		86.30 ± 8.02	
		On-edge, 29.68% FVF, 0.1mm layer thickness		246.19 ± 2.06	
		On-edge, 34.30% FVF, 0.1mm layer thickness		280.95 ± 3.77	
	Carbon + Nylon	Flat, 3.44%		22.21 ± 2.68	Flat: ductile fracture surface and better bond within layers.
		Flat, 24.94%		33.21 ± 0.94	
		Flat, 53.18%		57.50 ± 1.56	
	Kevlar + Nylon	Flat, 8.60%		30.11 ± 3.57	
		Flat, 29.50%		83.69 ± 6.10	
		Flat, 56.06%		125.47 ± 4.75	
	Glass + Nylon	Flat, 8.40%		74.16 ± 7.96	
		Flat, 29.15%		206.66 ± 2.27	
		Flat, 55.60%		271.19 ± 9.67	

**Table 2:** Infill pattern, fibre volume fraction (FVF), compressive strength and microstructural analysis details [175].

Printing Machine	Fibre + Matrix	Specimen Description	Test Specifications	Strength (MPa)	Modulus (GPa)	Microscope Analysis of Fracture Surface
Markforged- Mark Two	Carbon+ Nylon	Isotropic, 12 CF on top and 12 CF on bottom	Compressive-ASTM D695-15	30 ± 1.41	1.093 ± 0.019	Delamination occurred during compression. Height variation in fibre layers and nylon layers.
		Isotropic, 8 CF on top, 8 CF in middle, 8 CF on bottom		36.5 ± 2.12	1.332 ± 0.016	
		Isotropic, 24 CF equidistant layers		39.5 ± 0.707	1.475 ± 0.035	
		Concentric, 12 CF on top and 12 CF on bottom		30.5 ± 0.707	1.064 ± 0.021	Delamination occurred during compression. Height variation in fibre layers and nylon layers. Gaps between fibre concentric rings
		Concentric, 8 CF on top, 8 CF in middle, 8 CF on bottom		36 ± 0	1.472 ± 0.038	
		Concentric, 24 CF equidistant layers		40.5 ± 0.707	1.69 ± 0.005	
		Concentric, 24 CF equidistant layers, 8.18% FVF		40.4 ± 0.72	1.54 ± 0.04	Delamination occurred during compression. Height variation in fibre layers and nylon layers, but better adhesion of layers than other print orientations.
		Concentric, 24 CF equidistant layers, 16.59% FVF		43 ± 1.5	1.91 ± 0.09	
		Concentric, 24 CF equidistant layers, 24.44% FVF		53.3 ± 1.5	2.1 ± 0.04	
		Isotropic, perpendicular layers to load direction	Flexural-ASTM D790-10	38.89 ± 1.32	3.39 ± 0.14	
		Isotropic, parallel layers to load direction		26.91 ± 2.57	1.45 ± 0.04	
		Concentric, perpendicular layers to load direction		59.07 ± 2.10	5.41 ± 0.076	
		Concentric, parallel layers to load direction		35.38 ± 0.72	2.39 ± 0.05	
		24 CF equidistant layers, 17.18%		83.5 ± 5.49	5.16 ± 0.28	
		24 CF equidistant layers, 32.19%		143.3 ± 4.57	8.89 ± 0.39	
		24 CF equidistant layers, 48.93%		231.1 ± 15	14.17 ± 0.17	

**Table 3:** Infill pattern, strength, modulus and microstructural analysis details [183].

Printing Machine	Fibre + Matrix	Specimen Description	Test Specifications	Strength (MPa)	Microscope Analysis of Fracture Surface
Markforged- Mark Two	Carbon + Nylon	4 layers of, 4 concentric fibre rings with rectangular Nylon fill	Tensile test	72 ± 0.83	Better deposition of nylon matrix on carbon fibre pull-outs, indicating good adhesion between matrix and fibres compared to glass and Kevlar.
		6 layers of, 6 concentric fibre rings with rectangular Nylon fill		98 ± 2.92	
		6 layers of, 6 concentric fibre rings with hexagonal Nylon fill		83 ± 0.53	
		6 layers of, 6 concentric fibre rings with triangular Nylon fill		88 ± 0.33	
		8 layers of, 8 concentric fibre rings with rectangular Nylon fill		110 ± 2.09	
	Glass + Nylon	4 layers of, 4 concentric fibre rings with rectangular Nylon fill		48 ± 2.45	Nylon slightly deposited on the glass fibre pull-outs.
		6 layers of, 6 concentric fibre rings with rectangular Nylon fill		81 ± 1.80	
		6 layers of, 6 concentric fibre rings with hexagonal Nylon fill		70 ± 3.17	
		6 layers of, 6 concentric fibre rings with triangular Nylon fill		75 ± 0.90	
		8 layers of, 8 concentric fibre rings with rectangular Nylon fill		91 ± 3.43	
	Kevlar + Nylon	4 layers of, 4 concentric fibre rings with rectangular Nylon fill		47 ± 3.82	The smooth surface of the Kevlar fibre pull-outs indicates poor adhesion between nylon matrix and Kevlar fibres.
		6 layers of, 6 concentric fibre rings with rectangular Nylon fill		66 ± 1.12	
		6 layers of, 6 concentric fibre rings with hexagonal Nylon fill		63 ± 1.04	
		6 layers of, 6 concentric fibre rings with triangular Nylon fill		56 ± 0.32	
		8 layers of, 8 concentric fibre rings with rectangular Nylon fill		75 ± 0.99	

**Table 4:** Details of ultrasound treatment on the FDM print part.

Ref.	Fibre + Matrix	Ultrasound (US) Machine Details	Frequency/Amplitude	Time/Speed	Effect of Ultrasound on the FDM Print
[253]	ABS	US is applied during printing. 1 Piezoelectric transducer mounted on printing platform.	27/10 μm		Surface quality improved; surface roughness value: 14.03; layer thickness reduced due to compression occurrence; staircase effect removed
			40/10 μm		Surface quality improved; surface roughness: 13.70; layer thickness reduced due to compression occurrence; staircase effect removed
			50/10 μm		Surface quality improved; surface roughness: 13.66; layer thickness reduced due to compression occurrence; staircase effect removed
[256]	ABS	US is applied during printing. A 40 kHz-piezoelectric bolt-clamped transducer connected to the heater block of the printhead using a stainless-steel rod.	34.4 kHz		Increased layer adhesion by 10%
[257]	ABS	US is applied to the completed print part. Ultrasonic horn in contact with the printed specimen with 3.5 kg/cm^2^ pressure, 0.35S delay time, 0.4s hold time	20 kHz	0.5 s	Tensile strength increased by 11.3%; Young’s modulus increased by 16.7%; surface has decreased roughness
[258]	ABS	US is applied to the completed print part. Ultrasonic horn in contact with the printed specimen with 3.5 kg/cm^2^ pressure, 0.49s delay time, 0.5s hold time	20 kHz	0.65 s/0.5 s	Bending strength increased by 10.8% in a 0.65 s treatment time; bending modulus increased by 12.5% in a 0.5 s treatment time; rasters totally fused together when pressure increased up to 5 kg/cm^2^
[260]	Continuous CF+ PLA	Ultrasound transducer is connected from a 10mm distance to the CF bundle immersed in PLA resin	40 μm	15mm/s	Tensile strength and flexural strength increased by 34% and 29%
